# *catena*-Poly[[(2,2′-bipyidine-2κ^2^ *N*,*N*′)-μ-cyanido-1:2κ^2^ *N*:*C*-dicopper(I)]-μ-bromido-[(2,2′-bipyidine-2κ^2^ *N*,*N*′)-μ-cyanido-1:2κ^2^ *N*:*C*-dicopper(I)]-μ-cyanido-κ^2^ *N*:*C*]

**DOI:** 10.1107/S1600536812018120

**Published:** 2012-04-28

**Authors:** Jia Gu, Xuee Jiang, Zhanhua Su, Baibin Zhou

**Affiliations:** a Colleges of Chemistry & Chemical Engineering, Harbin Normal University, Harbin 150025, People’s Republic of China

## Abstract

In the title complex, [Cu_4_Br(CN)_3_(C_10_H_8_N_2_)_2_]_*n*_, the four independent Cu^I^ atoms are all in distorted trigonal-planar geometries. One is formed by one N atom and one C atom from two cyanide groups and one Br atom, one is formed by two N atoms from two cyanide groups and one Br atom, and the other two are formed by two N atoms from a chelating 2,2′-bipyridine (bpy) ligand and one C atom from a cyanide group. The structure exhibits a zigzag chain backbone along [101] constructed by bromide and cyanide anions bridging the Cu^I^ atoms, with the [Cu(bpy)(CN)] units pointing laterally.

## Related literature

For copper cyanide coordination polymers, see: Korzeniak *et al.* (2005 ▶); Yi *et al.* (2004 ▶). For structures containing cyanide groups, see: Zhang *et al.* (2000 ▶). For related copper complexes, see: He *et al.* (2006 ▶).
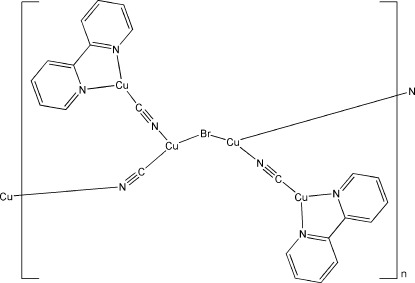


## Experimental

### 

#### Crystal data

[Cu_4_Br(CN)_3_(C_10_H_8_N_2_)_2_]
*M* *_r_* = 724.53
Monoclinic, 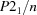

*a* = 10.0074 (10) Å
*b* = 17.7556 (17) Å
*c* = 14.5125 (14) Å
β = 102.924 (1)°
*V* = 2513.4 (4) Å^3^
*Z* = 4
Mo *K*α radiation
μ = 4.96 mm^−1^
*T* = 273 K
0.24 × 0.24 × 0.22 mm

#### Data collection

Bruker APEXII CCD diffractometer
Absorption correction: multi-scan (*SADABS*; Bruker, 2001 ▶) *T*_min_ = 0.383, *T*_max_ = 0.409
23142 measured reflections
6171 independent reflections
3491 reflections with *I* > 2σ(*I*)
*R*_int_ = 0.035

#### Refinement

*R*[*F* ^2^ > 2σ(*F* ^2^)] = 0.035
*wR*(*F* ^2^) = 0.084
*S* = 1.00
6171 reflections
317 parameters
H-atom parameters constrained
Δρ_max_ = 0.37 e Å^−3^
Δρ_min_ = −0.41 e Å^−3^

### 

Data collection: *APEX2* (Bruker, 2007 ▶); cell refinement: *SAINT* (Bruker, 2007 ▶); data reduction: *SAINT*; program(s) used to solve structure: *SHELXS97* (Sheldrick, 2008 ▶); program(s) used to refine structure: *SHELXL97* (Sheldrick, 2008 ▶); molecular graphics: *SHELXTL* (Sheldrick, 2008 ▶) and *DIAMOND* (Brandenburg, 1999 ▶); software used to prepare material for publication: *SHELXTL*.

## Supplementary Material

Crystal structure: contains datablock(s) I, global. DOI: 10.1107/S1600536812018120/hy2536sup1.cif

Structure factors: contains datablock(s) I. DOI: 10.1107/S1600536812018120/hy2536Isup2.hkl

Additional supplementary materials:  crystallographic information; 3D view; checkCIF report

## Figures and Tables

**Table 1:** Selected bond lengths (Å)

Cu1—N1	2.025 (3)
Cu1—N2	2.029 (3)
Cu1—C21	1.836 (4)
Cu2—N3	2.055 (3)
Cu2—N4	2.010 (3)
Cu2—C23	1.836 (3)
Cu3—N5	1.876 (3)
Cu3—Br1	2.5163 (6)
Cu3—C22	1.842 (3)
Cu4—N6^i^	1.906 (3)
Cu4—N7	1.878 (3)
Cu4—Br1	2.4650 (6)

Symmetry code: (i) 
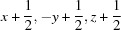
.

**Fig. 1.**
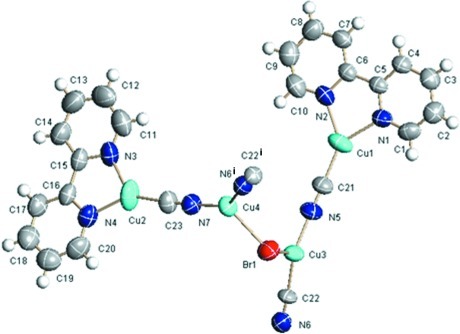
The asymmetric unit of the title complex, showing the coordination environments around the CuI atoms. Displacement ellipsoids are drawn at 50% probability level. [Symmetry code: (i) x+1/2, -y+1/2, z+1/2.]

**Fig. 2.**
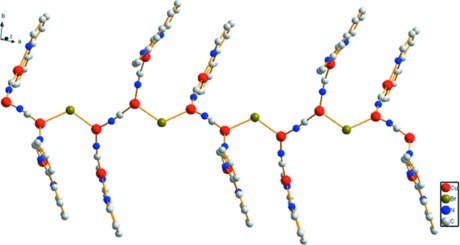
The zigzag chain in the title complex.

## supplementary crystallographic information

### Comment

Considerable attention has been paid to the study of copper cyanide coordination polymers due to their fascinating structural frameworks, physical and chemical properties, and potential applications in many fields (Korzeniak *et al.*, 2005; Yi *et al.*, 2004). Cyanide group is a versatile ligand that can act as a monodentate ligand as well as a µ_2_-, µ_3_- or µ_4_-bridging ligand, exhibiting intriguing topological architectures (Zhang *et al.*, 2000). Copper atom has versatile coordination properties and normally adopts two-, three-, four-, five-, or six-coordination, forming diverse geometries (He *et al.*, 2006). Herein, we report a copper cyanide coordination polymers derived from 2,2'-bipyidine ligand. The title complex contains four unique Cu^I^ ions, which are all in distorted trigonal-planar geometries. However, the detailed coordination environments of these Cu^I^ atoms are different, as Cu1 and Cu2 are each coordinated by two N atoms of a 2,2'-bipyidine ligand and one µ_2_-cyanide group. Cu3 and Cu4 are each coordinated by two µ_2_-cyanide groups and one bromide ion (Fig. 1, Table 1). The structure exhibits by a zigzag chain backbone, [Cu_2_Br(CN)]_n_, along [1 0 1] (Fig. 2). The [Cu(bpy)(CN)] units point lateral of the chain.

### Experimental

A mixture of CuBr_2_ (0.33 g, 1.48 mmol), K_4_Fe(CN)_6_.3H_2_O (0.42 g, 0.99 mmol), 2,2'-bipyridine (0.156 g, 1.00 mmol) and 24 ml H_2_O was stirred for 30 min in air. The resulting gel was then transferred to a 30 ml Teflon-lined autoclave and kept at 160°C for 5 days. After the mixture was slowly cooled to room temperature, yellow block crystals of the title complex were filtered, washed with water and dried at room temperature (yield: 0.17 g, 66%).

### Refinement

H atoms were positioned geometrically and refined as riding atoms, with C—H = 0.93 Å and *U*_iso_(H) = 1.2*U*_eq_(C).

### Crystal data

**Table:**

[Cu_4_Br(CN)_3_(C_10_H_8_N_2_)_2_]	*F*(000) = 1416
*M**_r_* = 724.53	*D*_x_ = 1.915 Mg m^−^^3^
Monoclinic, *P*2_1_/*n*	Mo *K*α radiation, λ = 0.71073 Å
Hall symbol: -P 2yn	Cell parameters from 4357 reflections
*a* = 10.0074 (10) Å	θ = 2.3–22.8°
*b* = 17.7556 (17) Å	µ = 4.96 mm^−^^1^
*c* = 14.5125 (14) Å	*T* = 273 K
β = 102.924 (1)°	Block, yellow
*V* = 2513.4 (4) Å^3^	0.24 × 0.24 × 0.22 mm
*Z* = 4	

### Data collection

**Table:**

Bruker APEXII CCD diffractometer	6171 independent reflections
Radiation source: fine-focus sealed tube	3491 reflections with *I* > 2σ(*I*)
Graphite monochromator	*R*_int_ = 0.035
φ and ω scans	θ_max_ = 28.3°, θ_min_ = 2.3°
Absorption correction: multi-scan (*SADABS*; Bruker, 2001)	*h* = −13→13
*T*_min_ = 0.383, *T*_max_ = 0.409	*k* = −21→23
23142 measured reflections	*l* = −19→19

### Refinement

**Table:**

Refinement on *F*^2^	Secondary atom site location: difference Fourier map
Least-squares matrix: full	Hydrogen site location: inferred from neighbouring sites
*R*[*F*^2^ > 2σ(*F*^2^)] = 0.035	H-atom parameters constrained
*wR*(*F*^2^) = 0.084	*w* = 1/[σ^2^(*F*_o_^2^) + (0.0336*P*)^2^ + 0.2889*P*] where *P* = (*F*_o_^2^ + 2*F*_c_^2^)/3
*S* = 1.00	(Δ/σ)_max_ < 0.001
6171 reflections	Δρ_max_ = 0.37 e Å^−^^3^
317 parameters	Δρ_min_ = −0.41 e Å^−^^3^
0 restraints	Extinction correction: *SHELXL97* (Sheldrick, 2008), Fc^*^=kFc[1+0.001xFc^2^λ^3^/sin(2θ)]^-1/4^
Primary atom site location: structure-invariant direct methods	Extinction coefficient: 0.00042 (11)

### Special details

**Table:**

Geometry. All e.s.d.'s (except the e.s.d. in the dihedral angle between two l.s. planes) are estimated using the full covariance matrix. The cell e.s.d.'s are taken into account individually in the estimation of e.s.d.'s in distances, angles and torsion angles; correlations between e.s.d.'s in cell parameters are only used when they are defined by crystal symmetry. An approximate (isotropic) treatment of cell e.s.d.'s is used for estimating e.s.d.'s involving l.s. planes.
Refinement. Refinement of *F*^2^ against ALL reflections. The weighted *R*-factor *wR* and goodness of fit *S* are based on *F*^2^, conventional *R*-factors *R* are based on *F*, with *F* set to zero for negative *F*^2^. The threshold expression of *F*^2^ > σ(*F*^2^) is used only for calculating *R*-factors(gt) *etc*. and is not relevant to the choice of reflections for refinement. *R*-factors based on *F*^2^ are statistically about twice as large as those based on *F*, and *R*- factors based on ALL data will be even larger.

### Fractional atomic coordinates and isotropic or equivalent isotropic displacement parameters (Å^2^)

**Table:**

	*x*	*y*	*z*	*U*_iso_*/*U*_eq_	
Cu1	0.35793 (5)	0.01457 (3)	0.84772 (3)	0.08134 (17)	
Cu2	1.10313 (5)	0.03539 (3)	0.62780 (4)	0.1015 (2)	
Cu3	0.46792 (5)	0.19932 (3)	0.63059 (3)	0.07283 (15)	
Cu4	0.87398 (4)	0.19909 (2)	0.81033 (3)	0.06160 (13)	
Br1	0.66910 (4)	0.26922 (3)	0.72846 (3)	0.08278 (14)	
N1	0.2531 (3)	0.01213 (16)	0.95197 (18)	0.0624 (7)	
N2	0.4332 (3)	−0.08313 (16)	0.91135 (18)	0.0633 (7)	
N3	1.1656 (3)	−0.07460 (17)	0.6509 (2)	0.0754 (8)	
N4	1.2024 (3)	0.02421 (17)	0.5226 (2)	0.0719 (8)	
N5	0.4205 (3)	0.12318 (17)	0.70702 (19)	0.0722 (8)	
N6	0.4104 (3)	0.26904 (16)	0.4389 (2)	0.0695 (8)	
N7	0.9502 (3)	0.13623 (16)	0.73146 (19)	0.0681 (7)	
C1	0.1708 (4)	0.0657 (2)	0.9725 (3)	0.0736 (10)	
H1	0.1423	0.1037	0.9284	0.088*	
C2	0.1261 (4)	0.0676 (2)	1.0554 (3)	0.0766 (10)	
H2	0.0695	0.1061	1.0674	0.092*	
C3	0.1669 (4)	0.0114 (2)	1.1194 (3)	0.0718 (10)	
H3	0.1379	0.0109	1.1760	0.086*	
C4	0.2521 (3)	−0.04517 (19)	1.0997 (2)	0.0605 (8)	
H4	0.2813	−0.0836	1.1431	0.073*	
C5	0.2931 (3)	−0.04371 (18)	1.0149 (2)	0.0535 (8)	
C6	0.3839 (3)	−0.10117 (18)	0.9871 (2)	0.0550 (8)	
C7	0.4153 (3)	−0.1688 (2)	1.0339 (2)	0.0668 (9)	
H7	0.3800	−0.1803	1.0863	0.080*	
C8	0.4990 (4)	−0.2189 (2)	1.0022 (3)	0.0851 (12)	
H8	0.5201	−0.2650	1.0324	0.102*	
C9	0.5519 (4)	−0.2003 (3)	0.9248 (3)	0.0854 (12)	
H9	0.6096	−0.2331	0.9022	0.102*	
C10	0.5169 (4)	−0.1325 (3)	0.8830 (3)	0.0805 (11)	
H10	0.5533	−0.1196	0.8315	0.097*	
C11	1.1467 (4)	−0.1211 (3)	0.7194 (3)	0.0967 (14)	
H11	1.0891	−0.1057	0.7580	0.116*	
C12	1.2070 (5)	−0.1896 (3)	0.7354 (3)	0.1034 (15)	
H12	1.1926	−0.2199	0.7845	0.124*	
C13	1.2888 (5)	−0.2128 (3)	0.6784 (4)	0.1056 (15)	
H13	1.3310	−0.2597	0.6873	0.127*	
C14	1.3087 (4)	−0.1667 (2)	0.6077 (3)	0.0893 (12)	
H14	1.3652	−0.1821	0.5684	0.107*	
C15	1.2458 (3)	−0.0972 (2)	0.5940 (3)	0.0633 (9)	
C16	1.2631 (3)	−0.0431 (2)	0.5204 (2)	0.0617 (9)	
C17	1.3361 (4)	−0.0580 (2)	0.4522 (3)	0.0811 (11)	
H17	1.3769	−0.1049	0.4504	0.097*	
C18	1.3487 (4)	−0.0046 (3)	0.3876 (3)	0.0975 (13)	
H18	1.3981	−0.0147	0.3418	0.117*	
C19	1.2890 (4)	0.0634 (3)	0.3904 (3)	0.0954 (12)	
H19	1.2972	0.1009	0.3473	0.114*	
C20	1.2164 (4)	0.0755 (2)	0.4580 (3)	0.0903 (12)	
H20	1.1742	0.1221	0.4593	0.108*	
C21	0.3923 (3)	0.0802 (2)	0.7580 (2)	0.0658 (9)	
C22	0.4283 (3)	0.24393 (18)	0.5130 (2)	0.0536 (8)	
C23	1.0047 (4)	0.09831 (19)	0.6881 (3)	0.0706 (10)	

### Atomic displacement parameters (Å^2^)

**Table:**

	*U*^11^	*U*^22^	*U*^33^	*U*^12^	*U*^13^	*U*^23^
Cu1	0.0947 (3)	0.0914 (4)	0.0562 (3)	−0.0338 (3)	0.0130 (2)	0.0137 (2)
Cu2	0.0880 (3)	0.0867 (4)	0.1408 (5)	0.0024 (3)	0.0487 (3)	−0.0510 (3)
Cu3	0.0990 (3)	0.0733 (3)	0.0532 (3)	0.0211 (2)	0.0318 (2)	0.0187 (2)
Cu4	0.0725 (3)	0.0637 (3)	0.0520 (2)	−0.0044 (2)	0.02109 (19)	−0.0135 (2)
Br1	0.0697 (2)	0.0949 (3)	0.0768 (3)	0.0063 (2)	0.00151 (19)	0.0136 (2)
N1	0.0643 (16)	0.0648 (19)	0.0558 (17)	−0.0068 (14)	0.0085 (13)	0.0070 (15)
N2	0.0630 (16)	0.073 (2)	0.0557 (17)	−0.0154 (14)	0.0176 (13)	−0.0033 (15)
N3	0.0662 (17)	0.072 (2)	0.095 (2)	−0.0112 (15)	0.0324 (17)	−0.0206 (18)
N4	0.0713 (18)	0.056 (2)	0.088 (2)	0.0030 (15)	0.0171 (16)	−0.0208 (17)
N5	0.093 (2)	0.075 (2)	0.0492 (17)	−0.0009 (16)	0.0170 (15)	0.0079 (15)
N6	0.0674 (18)	0.069 (2)	0.072 (2)	−0.0068 (14)	0.0151 (15)	−0.0024 (16)
N7	0.0760 (18)	0.0662 (19)	0.0627 (18)	0.0042 (15)	0.0169 (15)	−0.0111 (15)
C1	0.077 (2)	0.058 (2)	0.079 (3)	−0.0036 (19)	0.004 (2)	0.012 (2)
C2	0.078 (2)	0.071 (3)	0.078 (3)	0.001 (2)	0.011 (2)	−0.010 (2)
C3	0.078 (2)	0.080 (3)	0.059 (2)	−0.008 (2)	0.0191 (18)	−0.011 (2)
C4	0.066 (2)	0.063 (2)	0.052 (2)	−0.0099 (17)	0.0114 (16)	0.0017 (17)
C5	0.0533 (17)	0.057 (2)	0.0477 (19)	−0.0139 (15)	0.0061 (14)	0.0019 (16)
C6	0.0524 (17)	0.061 (2)	0.0503 (19)	−0.0149 (15)	0.0090 (14)	−0.0022 (16)
C7	0.066 (2)	0.066 (2)	0.070 (2)	−0.0063 (18)	0.0191 (17)	0.0082 (19)
C8	0.076 (3)	0.073 (3)	0.104 (3)	0.005 (2)	0.017 (2)	0.007 (2)
C9	0.076 (3)	0.088 (3)	0.095 (3)	−0.005 (2)	0.024 (2)	−0.022 (3)
C10	0.079 (3)	0.099 (3)	0.068 (3)	−0.019 (2)	0.027 (2)	−0.011 (2)
C11	0.082 (3)	0.108 (4)	0.108 (4)	−0.022 (3)	0.039 (3)	−0.016 (3)
C12	0.083 (3)	0.108 (4)	0.116 (4)	−0.020 (3)	0.015 (3)	0.026 (3)
C13	0.083 (3)	0.092 (4)	0.138 (4)	0.013 (2)	0.018 (3)	0.022 (3)
C14	0.078 (3)	0.082 (3)	0.108 (3)	0.015 (2)	0.021 (2)	−0.001 (3)
C15	0.0484 (17)	0.061 (2)	0.079 (2)	−0.0021 (16)	0.0100 (16)	−0.0222 (19)
C16	0.0492 (17)	0.061 (2)	0.071 (2)	0.0034 (16)	0.0059 (16)	−0.0193 (19)
C17	0.083 (3)	0.082 (3)	0.079 (3)	0.018 (2)	0.020 (2)	−0.014 (2)
C18	0.100 (3)	0.120 (4)	0.075 (3)	0.011 (3)	0.025 (2)	−0.009 (3)
C19	0.098 (3)	0.099 (4)	0.084 (3)	−0.005 (3)	0.011 (2)	0.005 (3)
C20	0.100 (3)	0.057 (3)	0.112 (4)	0.008 (2)	0.018 (3)	−0.008 (3)
C21	0.073 (2)	0.073 (2)	0.050 (2)	−0.0087 (18)	0.0102 (16)	0.0081 (18)
C22	0.0647 (19)	0.059 (2)	0.0359 (17)	−0.0024 (15)	0.0098 (14)	0.0112 (15)
C23	0.075 (2)	0.059 (2)	0.082 (3)	−0.0009 (18)	0.027 (2)	−0.019 (2)

### Geometric parameters (Å, º)

**Table:**

Cu1—N1	2.025 (3)	C4—C5	1.383 (4)
Cu1—N2	2.029 (3)	C4—H4	0.9300
Cu1—C21	1.836 (4)	C5—C6	1.480 (4)
Cu2—N3	2.055 (3)	C6—C7	1.381 (4)
Cu2—N4	2.010 (3)	C7—C8	1.370 (5)
Cu2—C23	1.836 (3)	C7—H7	0.9300
Cu3—N5	1.876 (3)	C8—C9	1.385 (5)
Cu3—Br1	2.5163 (6)	C8—H8	0.9300
Cu3—C22	1.842 (3)	C9—C10	1.357 (5)
Cu4—N6^i^	1.906 (3)	C9—H9	0.9300
Cu4—N7	1.878 (3)	C10—H10	0.9300
Cu4—Br1	2.4650 (6)	C11—C12	1.354 (6)
N1—C1	1.334 (4)	C11—H11	0.9300
N1—C5	1.347 (4)	C12—C13	1.351 (6)
N2—C10	1.339 (4)	C12—H12	0.9300
N2—C6	1.341 (3)	C13—C14	1.362 (5)
N3—C15	1.336 (4)	C13—H13	0.9300
N3—C11	1.338 (5)	C14—C15	1.379 (5)
N4—C20	1.337 (5)	C14—H14	0.9300
N4—C16	1.344 (4)	C15—C16	1.474 (5)
N5—C21	1.141 (4)	C16—C17	1.381 (4)
N6—C22	1.141 (4)	C17—C18	1.360 (5)
N7—C23	1.140 (4)	C17—H17	0.9300
C1—C2	1.374 (5)	C18—C19	1.351 (6)
C1—H1	0.9300	C18—H18	0.9300
C2—C3	1.363 (5)	C19—C20	1.363 (5)
C2—H2	0.9300	C19—H19	0.9300
C3—C4	1.387 (5)	C20—H20	0.9300
C3—H3	0.9300		
			
C21—Cu1—N1	138.90 (14)	C8—C7—C6	119.2 (3)
C21—Cu1—N2	139.10 (13)	C8—C7—H7	120.4
N1—Cu1—N2	80.99 (11)	C6—C7—H7	120.4
C23—Cu2—N4	146.27 (15)	C7—C8—C9	119.4 (4)
C23—Cu2—N3	132.60 (15)	C7—C8—H8	120.3
N4—Cu2—N3	81.13 (13)	C9—C8—H8	120.3
C22—Cu3—N5	145.80 (13)	C10—C9—C8	117.8 (4)
C22—Cu3—Br1	106.87 (10)	C10—C9—H9	121.1
N5—Cu3—Br1	107.33 (9)	C8—C9—H9	121.1
N7—Cu4—N6^i^	139.32 (12)	N2—C10—C9	124.0 (4)
N7—Cu4—Br1	114.45 (9)	N2—C10—H10	118.0
N6^i^—Cu4—Br1	106.16 (8)	C9—C10—H10	118.0
Cu4—Br1—Cu3	119.81 (2)	N3—C11—C12	123.4 (4)
C1—N1—C5	118.5 (3)	N3—C11—H11	118.3
C1—N1—Cu1	126.9 (2)	C12—C11—H11	118.3
C5—N1—Cu1	113.4 (2)	C13—C12—C11	118.4 (5)
C10—N2—C6	117.8 (3)	C13—C12—H12	120.8
C10—N2—Cu1	127.9 (2)	C11—C12—H12	120.8
C6—N2—Cu1	114.2 (2)	C12—C13—C14	119.3 (4)
C15—N3—C11	118.6 (3)	C12—C13—H13	120.4
C15—N3—Cu2	112.8 (3)	C14—C13—H13	120.4
C11—N3—Cu2	128.3 (3)	C13—C14—C15	120.5 (4)
C20—N4—C16	118.1 (3)	C13—C14—H14	119.7
C20—N4—Cu2	127.7 (3)	C15—C14—H14	119.7
C16—N4—Cu2	114.1 (3)	N3—C15—C14	119.8 (4)
C21—N5—Cu3	175.6 (3)	N3—C15—C16	115.9 (3)
C22—N6—Cu4^ii^	174.1 (3)	C14—C15—C16	124.3 (3)
C23—N7—Cu4	175.3 (3)	N4—C16—C17	120.1 (4)
N1—C1—C2	123.4 (3)	N4—C16—C15	115.8 (3)
N1—C1—H1	118.3	C17—C16—C15	124.1 (3)
C2—C1—H1	118.3	C18—C17—C16	120.4 (4)
C3—C2—C1	118.2 (4)	C18—C17—H17	119.8
C3—C2—H2	120.9	C16—C17—H17	119.8
C1—C2—H2	120.9	C19—C18—C17	119.5 (4)
C2—C3—C4	119.6 (3)	C19—C18—H18	120.3
C2—C3—H3	120.2	C17—C18—H18	120.3
C4—C3—H3	120.2	C18—C19—C20	118.4 (4)
C5—C4—C3	119.2 (3)	C18—C19—H19	120.8
C5—C4—H4	120.4	C20—C19—H19	120.8
C3—C4—H4	120.4	N4—C20—C19	123.5 (4)
N1—C5—C4	121.0 (3)	N4—C20—H20	118.2
N1—C5—C6	115.5 (3)	C19—C20—H20	118.2
C4—C5—C6	123.5 (3)	N5—C21—Cu1	175.3 (3)
N2—C6—C7	121.7 (3)	N6—C22—Cu3	175.7 (3)
N2—C6—C5	114.8 (3)	N7—C23—Cu2	175.0 (3)
C7—C6—C5	123.5 (3)		

Symmetry codes: (i) *x*+1/2, −*y*+1/2, *z*+1/2; (ii) *x*−1/2, −*y*+1/2, *z*−1/2.
